# Implementation of Maternal and Newborn Health Mobile Phone E-Cohorts to Track Longitudinal Care Quality in Low- and Middle-Income Countries

**DOI:** 10.9745/GHSP-D-23-00506

**Published:** 2024-08-27

**Authors:** Katherine Wright, Irene Mugenya, Emma Clarke-Deelder, Laura Baensch, Tefera Taddele, Anagaw Derseh Mebratie, Monica Chaudhry, Prashant Jarhyan, Nompumelelo Gloria Mfeka-Nkabinde, Jacinta Nzinga, Sailesh Mohan, Theodros Getachew, Margaret E. Kruk, Catherine Arsenault

**Affiliations:** a Department of Global Health and Population, Harvard T.H. Chan School of Public Health, Boston, MA, USA.; b Health Economics Research Unit, KEMRI Wellcome Trust Research Program, Nairobi, Kenya.; c Department of Epidemiology and Public Health, Swiss Tropical & Public Health Institute, Allschwil, Switzerland.; d Laterite, Nairobi, Kenya.; e Ethiopian Public Health Institute, Addis Ababa, Ethiopia.; f School of Public Health, College of Health Sciences, Addis Ababa University, Addis Ababa, Ethiopia.; g Public Health Foundation of India, Gurgaon, India.; h School of Nursing and Public Health, University of KwaZulu-Natal, Durban, South Africa.; i Department of Global Health, Milken Institute School of Public Health, George Washington University, Washington, DC, USA.

## Abstract

**Background:**

The maternal and newborn health (MNH) eCohort is a new mixed-mode (in-person and phone) longitudinal survey aiming to provide data on novel and undermeasured dimensions of quality along the MNH continuum of care. We describe implementation experiences and lessons learned in Ethiopia, India, Kenya, and South Africa to inform future longitudinal mobile phone-based studies on health system quality.

**Methods:**

To document the implementation approach and lesson learned, we engaged numerous stakeholders and conducted data reviews, debriefs, and a workshop with participants from all collaborative research organizations.

**Results:**

The MNH eCohorts enrolled women during their first antenatal care visit in 2 sentinel sites in Ethiopia, India, Kenya, and South Africa. In India, a site with better health outcomes and a site with poorer outcomes were chosen. In the remaining countries, an urban site and a rural site were chosen. Enrollment facilities reflect care-seeking patterns according to local health information data across public and private facilities and primary and secondary levels. Data collectors had a range of educational and experience profiles, and phone data collection was completed by the same enumerators in some countries and outsourced to data collection firms in others. Adequate infrastructure (including Internet and mobile phone coverage) was essential to implementation. Although follow-up is ongoing in India and South Africa, the eCohort retained 89%–90% of participants throughout the entire pregnancy and 78%–81% until 3 months postpartum in Ethiopia and Kenya, respectively.

**Conclusions:**

The MNH eCohort is a complex and long survey. Careful and thoughtful implementation demonstrates that it is a useful tool to gather data on health system quality and continuity and on changes in user experience over the continuum of care. Findings from the eCohort related to care and system competence and user experience will be valuable to program managers and policymakers alike.

## Abstract (precis)

We describe the feasibility, lessons learned, and challenges of implementing a longitudinal phone survey that followed women from their first antenatal care visit through delivery and until 3 months postpartum to assess health system competence, user experience, and health outcomes in Ethiopia, India, Kenya, and South Africa.

## Key Messages

- To address an important gap in longitudinal data on maternal and newborn health (MNH) care, the MNH eCohort—a longitudinal mixed-mode survey—was developed to measure quality of care, health system competence, and user experience, as well as link care processes to health outcomes.
- The MNH eCohort is a flexible and adaptable methodology that can be tailored to reflect local contexts, national priorities, and local best practices for data collection.
- In Ethiopia, data from the eCohort showed that during the first antenatal care visit, only 11% of women were screened for malnutrition, highlighting a potential area of improvement. In Kenya, women with risk factors received the same amount of care as women with no risk, and women who reported danger signs received the same amount of care as women not reporting danger signs, highlighting a need for better-targeted management of women at risk of poor pregnancy outcomes.
- Successful implementation of the eCohort methodology requires a carefully reviewed tool that reflects national programs and contexts, accurate programming of data collection instruments, sufficient human resources and infrastructure for data collection, a study population with access to a mobile phone, and adequate Internet and phone network coverage.

## BACKGROUND

Improving maternal and newborn health (MNH) remains a key global priority. To improve MNH, it is critical that women and their newborns receive high-quality care throughout pregnancy, delivery, and the postpartum period. Continuity of care is of particular importance for MNH, as complications that arise must be followed and managed appropriately.^1^ Existing data sources on quality MNH care, including Demographic and Health Surveys (DHS) and UNICEF’s Multiple Indicators Cluster Surveys, provide valuable data on health service utilization and often suggest poor quality care; however, the level of detail and information regarding service quality along the MNH continuum are limited. Longitudinal data on the amount, content, and quality of MNH care are scarce, and this information is critical for evidence-informed policies and programming for improved health outcomes.^2^ To address this important data gap, the MNH eCohort, a longitudinal mixed-mode (in-person and mobile phone) survey, was developed by the Quality Evidence for Health System Transformation (QuEST) Network, a multicountry consortium of health system researchers and policymakers.

The MNH eCohort provides prospective data on important processes of care measures and outcomes for women and their newborns. Women are enrolled in the cohort at their first antenatal care (ANC) visit and followed throughout their pregnancy and the postpartum period until 3 months after delivery. The MNH eCohort aims to measure care and system competence, user experience, and selected health outcomes (e.g., self-reported health, depression, and breastfeeding status) for mothers and newborns across the continuum spanning antenatal, intrapartum, and postnatal care. The MNH eCohort focuses on undermeasured dimensions of health system quality across this continuum. A more detailed description of the survey content, structure, development process, and its comparison to other existing tools will be published in a separate article.

The MNH eCohort focuses on undermeasured dimensions of health system quality across the care continuum.

Longitudinal surveys have many benefits, including reducing recall bias given the prospective nature and the real-time follow-up, establishing a sequence of events, and being able to follow changes over time in the study population. In particular, unlike cross-sectional surveys on MNH care quality, the eCohort will allow us to link processes of care to health outcomes and identify whether the health system interventions were effective in improving MNH outcomes and survival. Collecting longitudinal data is crucial to measure certain dimensions of quality, including the assessment of care continuity and timeliness of care, and to link care processes to health outcomes. The collection of longitudinal data for MNH is especially important, given the dynamic nature of pregnancy and the potential for potential health problems to arise at any time. The longitudinal nature of the study also allows the establishment of temporality and the performance of causal inference. These kinds of findings are essential for evidence-informed policies.

Conducting such studies by phone contains costs and can speed up data collection, though few people have tried to implement such a study in lower-income settings. As mobile phone access and ownership has increased, the use of mobile phones as a data collection tool has also increased.^3^ Mobile phones address many of the barriers seen in traditional survey tools; they are a way to rapidly collect data at a lower cost and with privacy for respondents. The use of mobile phones continues to be a promising approach.^4^^,^^5^

Starting in March 2023, the MNH eCohort was pretested and implemented for the first time in 2 sites each in Ethiopia, India, Kenya, and South Africa. In this article, we describe the implementation approach and lessons learned in these 4 countries. The MNH eCohorts built on previous experiences with mobile-phone data collection, and this article documents the decisions that research teams made during the implementation of this novel study.^3^^–^^5^ We aim to inform future implementation of the MNH eCohort as well as other future longitudinal mobile phone-based studies on health system quality in similar contexts.

## METHODS

The MNH eCohorts were implemented by research institutions based in each of the 4 countries. To facilitate successful study implementation in each country, research teams engaged numerous stakeholders, including national and subnational officials, professional bodies, technical working groups, non-state actors, academics, and facility and community health workers. In Ethiopia, this included both the Strategic Affairs and Community Engagement and Primary Health Care Lead Executive Offices at the Ministry of Health, as well as the Oromia Regional Health Bureau. In India, collaborators included many state (Haryana and Rajasthan) and district-level (Sonipat and Jodhpur) actors. In Kenya, teams collaborated with the Monitoring and Evaluation Department in the Ministry of Health, as well as the Kenya Medical Practitioners and Dentists Council. In South Africa, the primary health care transformation committee was an important stakeholder. These collaborators were involved in the eCohort design and identification of specific country-level priorities. In Ethiopia, priority areas of interest included maternal nutrition and utilization of maternity waiting homes. India’s eCohort emphasized chronic diseases in pregnancy; given the differences in health outcomes between the 2 sites, the eCohort aims to understand important differences in care across the sites. In Kenya, post-delivery discharge care patterns, adolescent health, psychosocial and emotional well-being, and the addition of key indicators to the Kenya Health Information System were priority focus areas. South Africa explored respectful intrapartum care, adolescent pregnancies, reasons for late initiation of ANC, and prevention of mother-to-child transmission of HIV. In all settings, the eCohort instruments were driven by local MNH guidelines, as well as World Health Organization global guidance.^6^^–^^9^ This demonstrates the utility of the eCohort and its responsiveness to local context and priorities. Throughout the implementation process, we used data reviews, team debriefs, and a workshop of all implementing organizations to document the implementation approach and lessons learned.

### The MNH eCohort Survey Methodology

The MNH eCohort survey includes 5 women survey modules: (1) a baseline in-person survey, (2) a repeated monthly phone survey module during pregnancy, (3) a phone survey after birth (2–4 weeks after birth), (4) a postnatal phone survey 6–8 weeks after birth, and (5) an endline in-person postnatal survey 10–12 weeks after birth. All phone surveys were computer-assisted telephone interviews, an interview technique where trained data collectors called respondents’ phone numbers and administered an interview following a script. Depending on gestational age at recruitment (at the first ANC visit), participation in the cohort lasts between 3 months (recruitment at 8 months’ gestation) and 10 months (recruitment at 2 months’ gestation). Women were enrolled in an in-person interview during their first ANC visit at a health facility and followed up by phone every 4 weeks during their pregnancy through 12 weeks postpartum. The endline survey was also an in-person survey.

Pre-tests were conducted in each country to identify methodological challenges, including with survey programming and data collection. Based on experiences and learnings from the pre-tests, implementation teams adjusted data collection tools and approaches in each of the 4 countries.

### Documentation of Lessons Learned

To effectively capture the lessons from eCohort design and implementation, we held debriefs with lead implementers and researchers in each country and data collectors working at the sites and in call centers. Table 1 describes the organizations implementing the eCohorts that were included in these debriefs. These debriefs documented challenges that the teams encountered as well as strategic decisions that were made to ensure successful data collection, minimize biases, and determine whether a mobile phone panel survey was a valid method to collect health system quality data.

**TABLE 1.** Debriefs to Document Experiences With Maternal and Newborn Health eCohort Implementation in Four Countries

	**Researchers/ Implementers**	**Data Collectors**
Ethiopia		
Ethiopian Public Health Institute	4 researchers	30
Addis Ababa University	1 researcher	
India		
Public Health Foundation of India	3 researchers	8
Kenya		
Kenya Medical Research Institute-Wellcome Trust	3 researchers	21
Laterite	2 implementers	17
South Africa		
University of KwaZulu Natal	3 researchers	6

Discussions with lead implementers documented challenges encountered and strategic decisions to ensure successful data collection and minimize biases.

We summarized findings from debriefs and reviewed data from pre-tests. When enrollment in the eCohorts in Ethiopia, Kenya, and South Africa was completed, representatives from each country (and India) attended a weeklong workshop to share best practices, lessons learned, and key changes to be made in future iterations of the eCohorts. Finally, data collection experiences from ongoing phone and endline surveys are also included. Information from all sources was triangulated and summarized to distill experiences, perspectives, and lessons learned.

### Ethical Approval

The study protocol was reviewed and approved by the Institutional Review Boards (IRB) of the Harvard T.H. Chan School of Public Health (protocol #IRB22-0487), the Kenya Medical Research Institute (protocol number KEMRI/SERU/CGMR-C/4226), the Ethiopian Public Health Institute (protocol number EPHI-IRB-448-2022), the University of KwaZulu-Natal (protocol number BREC/00004645/2022), and the Public Health Foundation of India (protocol number TRC-IEC 495/22).

## RESULTS

### Country-Specific Implementation Approaches

The MNH eCohort teams in each of the 4 implementing countries tailored the eCohort to that country’s context. Table 2 summarizes the country-level adaptations to the methodology regarding data collection strategies, enumerators, languages, incentives, and mobile phone distribution. Country teams reviewed all modules to ensure adherence to national guidelines and other contextual factors (using local language and phrases, standard treatment guidelines, and locally available medications).

**TABLE 2.** Country-Specific Adaptations to the MNH eCohort Implementation Approach

**Country**	**Priority Areas of Interest**	**Minimum Enrollment Age, Years**	**In-Person Enumerators for Baseline and Endline Modules**	**Phone Enumerators**	**Incentives – Phone Follow-up**	**Incentives – Endline**	**Maternal Card Review**
Ethiopia	Maternal malnutrition and mental health, and maternity waiting homes; added specific questions	15	30 hired by EPHI		50 birr airtime provided per month after completion of surveys	Wrapper for baby and maternal pad	Reviewed Integrated Maternal and Newborn Health card at baseline for general health history, obstetric history, content of care for ANC1
Kenya	Post-discharge care patterns; adolescent reproductive health; psychosocial and emotional well-being; key indicators to improve Kenya Health Information System	15	21 and 2 supervisors employed by KEMRI-Wellcome Trust	17 employed by Laterite	50 Kenyan shillings provided after completion of each module	Bar of soap	Reviewed Mother-Child Health Handbook at endline for delivery care and outcomes, postnatal care quality, and immunizations
India	Chronic diseases in pregnancy (including mental health, hypertension, and diabetes); sites have important differences in outcomes (one poor, one better), aim to understand if care is different between sites	18	8 (with ANM/staff nurse and/or master’s degree with relevant experience) hired by PHFI	8 (with ANM/staff nurse and/or master’s degree with relevant experience) hired by PHFI	669 Indian rupees (depending on service provider) of airtime provided every 3 months	Gift basket with items for basic newborn care	Reviewed Mother and Child Protection card at facility at baseline and endline
South Africa	Adolescent pregnancies, HIV/PMTCT, reasons for booking first ANC late in pregnancy, respectful intrapartum care	15 with parental consent or 18 otherwise	10 living in Nongoma and 7 in Umhlatuze, hired by UKZN	6 hired by UKZN based in Durban, South Africa	∼30 South African Rand provided after each completion of a phone survey	Additional airtime	Reviewed Road to Health Card at endline to collect information about infant’s health, including PMTCT

Abbreviations: ANC, antenatal care; ANM, auxiliary nurse-midwife; EPHI, Ethiopian Public Health Institute; KEMRI, Kenya Medical Research Institute; MNH, maternal and newborn health; PHFI, Public Health Foundation of India; PMTCT, prevention of mother-to-child transmission of HIV; UKZN, University of KwaZulu Natal.

### Selection of Implementation Sites

In each of the 4 countries, 2 sentinel sites were selected using a slightly different rationale (Table 3). The eCohort was not intended to be nationally representative. Instead, it aimed to provide actionable evidence for health system improvement in selected sites that represent a typology of health system challenges throughout the country. We initially aimed for 1 site to be predominantly urban and the other predominantly rural. In Kenya and South Africa, sites were selected based on poor MNH outcomes. In Ethiopia, health outcomes and prior relationships with health system actors, as well as availability of electricity and network coverage, determined the site selection. India chose 2 sites, including 1 with relatively better outcomes, to assess if care competence could explain differences in outcomes. Both sites in India include a mix of urban and rural.

**TABLE 3.** eCohort Data Collection Sites and Study Samples in Four Countries

	**Ethiopia**		**India**		**Kenya**		**South Africa**	
Implementation sites	Adama Town	East Shewa	Jodhpur	Sonipat	Kitui	Kiambu	Nongoma	Umhlathuze
eCohort enrollment dates	April 2023–May 2023		October 2023–January 2024^a^		June 2023–September 2023		April 2023–September 2023	
Survey administration languages	Amharic, Afan Oromo		Hindi		Kiswahili, Kamba, English		IsiZulu, English	
Administrative level	Zones in Oromia Region		District, State of Rajasthan	District, State of Haryana	County in the former Eastern Province	County in the former Central Province (located near the capital Nairobi)	Local municipality, Zululand district, Province of KwaZulu-Natal	Local municipality, King Cetshwayo District, Province of KwaZulu-Natal
Total population, no.	222,035^a^	1,357,522^a^	4,720,000	1,640,000	1,136,187	2,417,735	212,000	450,000
Area, km^2^	29.86	8,371.0	22,850.0	2,122.0	30,496.4	2,543.5	2,182.0	1,233.0
Population density	7,435.9	162.2	206.5	772.8	37.3	950.6	97.2	365.0
Rurality/ classification	Predominantly urban	Predominantly rural	34.3% urban 65.7% rural	31.3% urban 68.7% rural	Rural (arid and semi-arid land)	Urban	Rural	Urban
Maternal mortality ratio, per 100,000 live births	n/a	n/a	170	67	67	78	n/a	191
Recruitment, women (facilities), no.								
Public primary	257 (3)^b^	360 (8)^b^	271 (10)^c^	345 (11)^c^	295 (6)^d^	302 (3)^d^	516 (9)	528 (13)
Public secondary	48 (2)	37 (2)	231 (5)^c^	175 (3)^c^	108 (2)^d^	134 (2)^d^	–	–
Private	187 (6)^b^	111 (1)^b^	–	–	101 (4)^d^	62 (4)^d^	–	–

Abbreviation: n/a, not available.

^a^ Summary and Statistical Report of the 2007 Population and Housing Census, Population by Age and Sex. Federal Democratic Republic of Ethiopia Population Census Commission, December 2008, Addis Ababa.

^b^ In Ethiopia, public primary sites include public health centers, and private primary sites include private health centers and private maternal and child health clinics/centers.

^c^ In India, primary facilities include primary health centers and subcenters, and secondary facilities include district hospitals and community health centers.

^d^ In Kenya, public primary sites include government dispensaries and health centers, and private primary sites include private clinics, faith-based organization (FBO) dispensaries, and FBO health centers. Private secondary includes FBO and private hospitals.

### Sampling and Recruitment

We aimed to recruit women who were representative of health system users in the selected sites. This was achieved through the selection of facilities included as enrollment sites, as well as the decisions (discussed in later sections) regarding the distribution of mobile phones to women participating in the eCohort. A total sample of approximately 1,000 women was recruited per country, with 500 women enrolled in each of the 2 sites. Women were approached for enrollment in the MNH eCohort if they presented for their first ANC visit at an enrolling health facility during the data collection enrollment period. Eligibility criteria included: (1) being at least age 15 years in Ethiopia and Kenya, 18 years in India, or 15 years in South Africa with parental consent to participate); (2) being pregnant and attending their first ANC visit; (3) planning to continue to receive maternal health care in the same subnational area; and (4) being willing to be contacted on the phone every month for the duration of the study. Exclusion criteria included being at the health facility for a follow-up/subsequent ANC visit; being at the health facility for another health concern or to accompany someone else; and any physical, mental, or cognitive disability that would prohibit the ability to provide informed consent. Sample size justification is provided elsewhere.

Within each site, a master facility list—including private facilities—was obtained, and facilities were stratified by type, including public primary facilities, public secondary (hospitals), private primary facilities (e.g., clinics, maternal and child health [MCH] specialty centers or MCH specialty clinics), and private hospitals. In each site, we also obtained data on the volume of ANC visits by facility type (using DHS or health management information system data). Care-seeking patterns across the 4 strata (i.e., proportion of women who use public primary, public secondary, private primary, or private secondary facilities for ANC) dictated the sampling strategy. We aimed to include health facilities that would be representative of ANC utilization patterns at the site. The number and types of facilities selected for enrollment are described in Table 3.

In India and South Africa, private-sector facilities were excluded due to refusal to participate in the study. In Ethiopia, 2 private facilities, 1 MCH center, and 1 hospital were dropped due to a lack of ANC first clients for the first week of data collection. Enrolling women at the secondary level was also limited, given the fact that few women initiate care for ANC at the secondary level but may be referred to higher-level care throughout their pregnancy.

Collaboration with subnational authorities and the facility in-charge was an important step to ensure participation of health facilities as enrollment sites. In all sites, inception visits with the subnational authorities and at selected health facilities built trust and fostered collaboration.

### Data Collection

The mixed-mode nature of the eCohort requires 2 distinct types of data collection: in-person data collection at baseline and endline and computer-assisted telephone interviewing for the rest of the survey.^10^ The number and background of enumerators employed for the in-person vs. phone data collection varied across the 4 countries (Table 2). All data collectors had a minimum of secondary-level education, had previous data collection experience, and were fluent in all necessary local languages. In Ethiopia and India, data collectors had a degree in health, which includes nursing or master’s degrees, though such advanced degrees were not a requirement. In Kenya, in-person data collectors had nursing degrees, and phone-based data collectors were required to have a bachelor’s degree. In South Africa, data collectors for in-person surveys resided in the selected sites.

The mixed-mode nature of the eCohort requires both in-person data collection at baseline and endline and computer-assisted telephone interviewing for the rest of the survey.

### In-Person Recruitment

Mainstage data collection began in May 2023 in Ethiopia, June 2023 in Kenya, May 2023 in South Africa, and October 2023 in India. Data collectors were assigned to participating health facilities to enroll women and complete the baseline survey. During the enrollment period, they were posted to a health facility and enrolled eligible women until the desired number of women at each health facility (based on ANC volume and DHS utilization estimates) was reached.

In-person data collection for the baseline survey was needed to identify participants, collect data on the health facilities where women were recruited, and establish a relationship between the study and the participants. The in-person recruitment also included health assessments (described later) and a review of health cards. During this initial contact, data collectors explained and answered questions about the study, obtained informed consent, and distributed phones to women.

A central component of the in-person data collection was to gather sufficient information to allow tracing of enrolled women during the phone follow-up. As part of the baseline interview, data collectors gathered as many phone numbers as possible from each woman, as well as information about her residence. On average, women provided 1.6 phone numbers in Ethiopia and 2.7 in Kenya. Additional phone numbers may have been a secondary phone that she owned, a partner’s phone, or a friend/neighbor’s phone. During the in-person baseline, data collectors also established a preferred time of day for follow-up phone calls.

#### Mobile Phone Distribution

To ensure that women could be reached throughout the study period, mobile phones were an essential data collection tool, and all women enrolled in the study had to have access to a mobile phone. While mobile phone penetration continues to increase with time, ensuring representation of all system users (including those without mobile phones) necessitated the distribution of phones to women enrolling in the cohort, preventing systematically leaving out women without phones.^3^^,^^10^^–^^12^ The MNH eCohort distributed phones as part of the baseline enrollment. Phones were acceptable makes and models and included all necessary accessories (chargers, airtime, and SIM cards). Provision of phones is emerging as a best practice, and the distribution of these phones can be tailored to meet the unique context of each country.^3^

While mobile phone penetration continues to increase, ensuring representation of all users necessitated distributing phones to women enrolling in the cohort.

In all 4 countries, women were offered phones at enrollment. If they did not want a phone, they were offered an equivalent amount of airtime to be used on their existing phone lines. For example, in Ethiopia and Kenya, 97.9% and 93.4% of women accepted phones, respectively, while only around 4% of women took phones in South Africa. To date, there is very little evidence of phones being lost, stolen, or broken in the eCohort. However, there was anecdotal experience from Kenya where women did not have ownership of study phones, and approval from partners or husbands was necessary before women could accept the phone or agree to participate in the study. This made it difficult to reach a few women. This study clearly demonstrates that distributing mobile phones as a data collection tool is a valid approach despite these constraints.

#### Health Assessments

The MNH eCohort performed a series of health assessments at baseline to better assess the health of women enrolled. These assessments included height, weight, blood pressure, and hemoglobin measurement. In Ethiopia, the mid-upper arm circumference, which is the recommended method for identifying maternal malnutrition, was also measured. All measures, except for hemoglobin measurement, were repeated even if taken during the ANC visit. Women’s ANC records were consulted, and if the ANC visit included a hemoglobin test, that number was recorded. If the number was not available, a hemoglobin test was conducted by eCohort data collectors. The data collectors without medical training received sufficient training to collect blood pressure and hemoglobin measurements to successfully collect these important data.

#### Maternal Health Card Review

Data extraction from source documents was the final method of data collection for the eCohort. This extraction served to validate women’s self-report, compare what women knew about their health and what the health system knew, and assess recordkeeping throughout ANC visits and the intrapartum period, as well as neonatal outcomes.^13^ In Kenya, South Africa, and India, women carried maternal health card booklets with them that data collectors could review during enrollment interviews. In Ethiopia, these records remained at the facility, so data collectors reviewed them and then returned them to facility staff. In South Africa, more detailed information on delivery records was desired, so study teams assessed the feasibility of getting data from delivery facilities. A key challenge experienced in South Africa was the lack of data often recorded in these records. Many data fields were blank, making validation of self-report difficult.

### Mobile Phone Follow-Up and Call Tracing

Between the in-person baseline and endline surveys, women were followed up by phone calls made at approximately monthly intervals. These interim surveys aimed to ask participants about additional care that they had received, the content of that care, the women’s perception of that care, and their health status. For the purposes of the MNH eCohort, computer-assisted telephone interviews were the preferred data collection approach because it helped retain women in the cohort by sustaining rapport and relationships, facilitated participation by all women (including women who were illiterate, compared to text message responses), and ensured the accuracy of data collection (compared to interactive voice response). Building rapport with participants could also be achieved by having the same data collector call participants over the course of the study, which also appeared to have improved retention—a strategy employed by Kenya, Ethiopia, and South Africa to date. At the end of each call, data collectors confirmed the preferred phone number and time for the follow-up call. The preferred time of call for women changed throughout the duration of this study. In the calls during her pregnancy, she may be working, studying, or carrying out other duties, but after the birth of the baby, the preferred time of call was likely to shift. Data collectors were responsive to these changes in the demands of the participants’ time and schedule and conducted calls at a convenient time for participants.

Data collectors followed clear procedures during phone data collection (Box). Previous experience with phone surveys indicates collecting multiple phone numbers is a best practice. In the experience of the MNH eCohort, women were typically reached on their primary phone number, which the woman owned, and frequent calling of additional numbers was seen as a nuisance. However, in the unfortunate event of a maternal death, which occurred 3 times so far, those were identified by calling additional phone numbers (of husbands or sisters).

BOX Call Tracing Procedures

1. Four weeks after enrollment, data collectors call the primary phone number at the agreed-upon time to administer Module 2.
2. If no answer, call each additional number provided until they reach the respondent.
3. If not reached on Day 1, call all phone numbers 1 time each day for 7 days, varying call time on later days.
4. If reached on a non-primary number, confirm if the preferred number/time of call has changed.
5. If not reached after 7 days, trace her from the facility.
6. If not contacted in 2 consecutive months, women noted as lost to follow-up.

If a woman was reached on a number that was not noted as her primary phone number, data collectors confirmed if the preferred number and time of day had changed. If no contact was made with the woman in a particular month, she was noted as missed for that month, and data collectors attempted to call again 1 month from the date of the first call made. Women were considered lost-to-follow-up in the eCohort if they went 2 months without any contact, and data collectors stopped attempting to call that woman.

The phone-based data collection was an important component of the eCohort and required substantial infrastructure to carry out the many calls required in this study. In India and Ethiopia, the eCohort used an existing call center within the research team. In South Africa, phone data collection infrastructure was established for the first time. In Kenya, phone data collection was contracted out to an external firm. We found that having prior experience with phone-based data collection was very helpful for smooth implementation of the eCohort. Phone-based data collection required significant investment in infrastructure (phone, computers, headsets, staff, integrated call management systems, and Internet), quality control systems, and staff capacity. Call center operations also relied on strong power and network connections, and if those failed, interruptions to study calls occurred. In the event of these interruptions, alternative methods for calls (i.e., using mobile phones) were used.

The phone-based data collection was an important component of the eCohort and required substantial infrastructure to carry out the many calls required in this study.

### Attrition Management and Incentives

To ensure ongoing participation in the study, it was critical for participants to have sufficient airtime to keep their phone lines open and able to receive calls.^10^ Different incentive approaches were applied by each country regarding airtime (Table 2), but generally, each country credited each woman with a small amount of airtime upon completion of each phone interview. In Kenya, if a woman did not respond to the calls, a text message reminding her of the study interview and airtime top-up was sent, which often resulted in a completed survey.

Ethiopia completed the eCohort in February 2024, and Kenya completed the eCohort in July 2024. In Ethiopia, of 1,000 women, 92% completed at least 1 instance of module 2 (additional ANC care during the antenatal period), 89% completed module 3 (delivery care and delivery outcomes), and 83% of women completed the endline survey that concludes participation in the eCohort (module 5). In Kenya, of 1,010 women, 90% completed at least 1 instance of module 2, 88% of women were followed throughout the pregnancy (module 3), and 86% of women completed the endline survey (Figure). Data collection in India and South Africa is ongoing.

**FIGURE:**
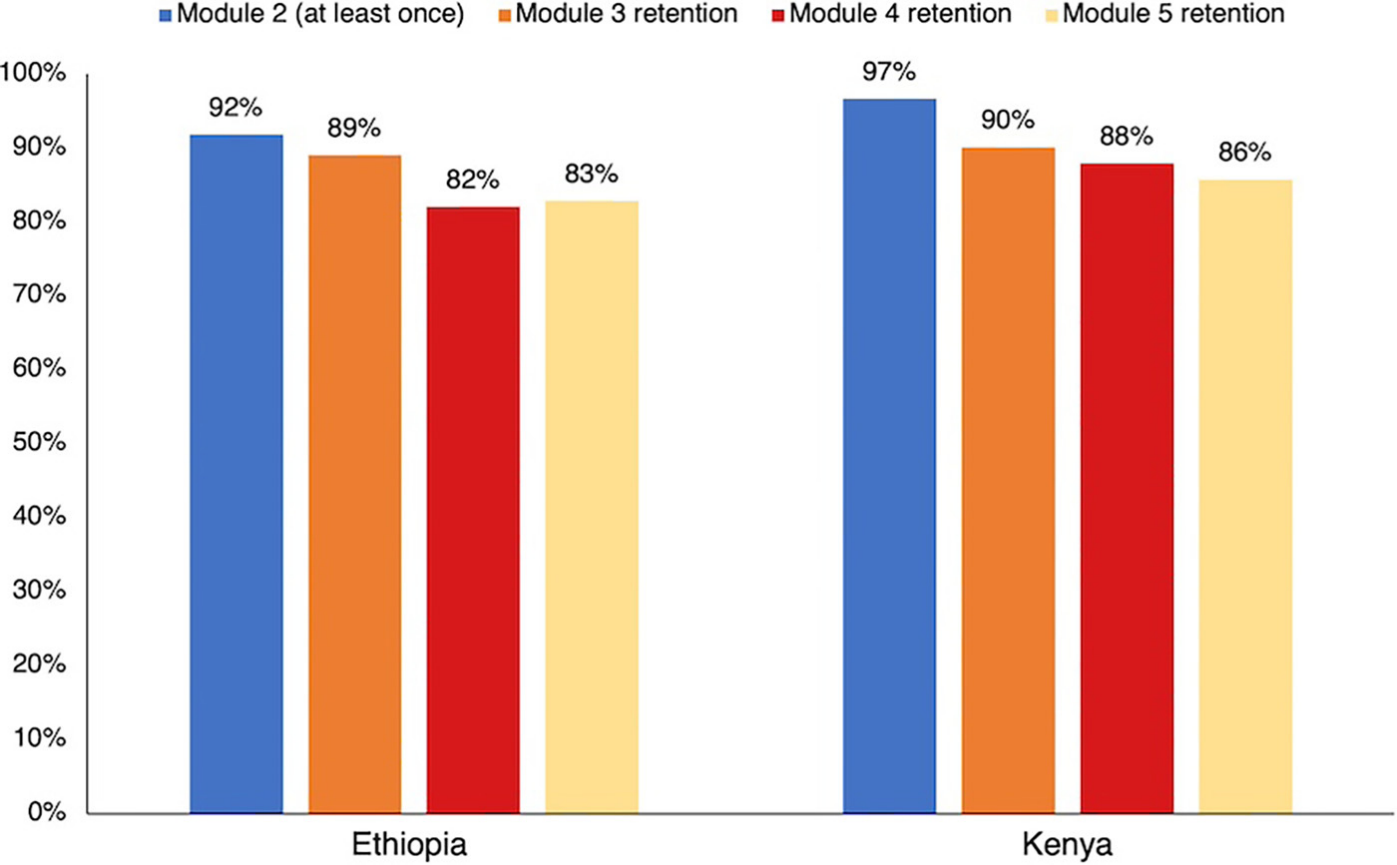
Percentage of Women Who Completed Modules of the MNH eCohort in Ethiopia and Kenya

Women who have dropped out of the study cite fatigue and that the calls take up too much time as reasons for dropping out. To date, refusals and loss-to-follow-up are not outside of the expected range.^14^ Calculating loss-to-follow-up with the study still ongoing is difficult but will be carefully analyzed upon completion of the cohorts and in all subsequent analyses.

Throughout the eCohort, pregnancy loss, neonatal death, and infant death were inevitable outcomes experienced. The eCohort defined stillbirths as fetal deaths that occurred at 28 weeks or more of gestation and miscarriages as fetal deaths that occurred before 28 weeks of gestation. Women who experienced these outcomes were asked to respond to a series of relevant questions (e.g., in case of stillbirth, questions focused on the content and quality of intrapartum care; in case of neonatal or infant death, questions covered additional care-seeking and cause of death). After experiencing any of these outcomes, women completed their study participation and were no longer contacted for further follow-up interviews. Retention rates reported here include these women who concluded their survey participation early. About 5% of women experience these outcomes; 54 women in Ethiopia and 55 women in Kenya ended their participation during rounds of module 2 or after module 3 after a miscarriage, stillbirth, or neonatal and infant death.

### In-Person Endline Survey

The in-person endline survey facilitated a second round of health assessments, including weight and head circumference measurements for the babies and repeats of the health assessments taken at baseline for the women. The in-person endline also concluded the women’s participation in the survey and provided an opportunity to distribute incentives. Endline surveys are currently ongoing, but contextual factors appear important to consider when planning the endline. For example, in Kenya, women typically returned to their maternal village after delivery and would be out of the study area during the endline data collection. In Ethiopia, confinement periods may have impacted the ability to carry out in-person endline data collection. Where baseline interviews took place at health facilities, endline surveys could take place in a more central location, not necessarily at the health facility, and on a more flexible schedule. To accommodate women who were out of the study period, endline surveys could also be conducted on the phone to gather a few final key measures and forego the health assessments.

### Procurement and Budget Implications

An understanding of the procurement and budgetary implications of this study is important. Knowing the cost drivers in these first 4 countries can help future implementers consider how the eCohort might be adapted or implemented in varying contexts with varying budgets and resources. In each of the 4 countries, the MNH eCohorts were completed with a budget of US$200,000. Funds were distributed from QuEST Harvard to our network partners in the study countries. Teams allocated the funds as they saw fit for their context. In Kenya, a data collection firm with experience administering phone-based surveys was hired to carry out all data collection except the baseline and endline surveys. Costs associated with analysis and dissemination are not included here.

Table 4 shows the budget breakdown across key categories, including staff to oversee study implementation (including programmers to set up data collection instruments and databases), data collector salaries, costs associated with in-person data collection, procurement (phones, tablets, data collection equipment, and incentives provided to women), costs associated with administering phone surveys (establishing a call center or contracting to a firm), phones/airtime, and other study requirements.

**TABLE 4.** MNH eCohort Data Collection Budget Breakdowns in Four Initial Implementing Countries

**Cost Category**	**%**			
	**Ethiopia**	**Kenya**	**South Africa**	**India**
Personnel (data collectors)	37	14	41	40
Data collection subcontract	n/a	50	n/a	n/a
Travel for supervision	23	10	15	8
Tool translation and programming	1	n/a	1	7
Workshops and trainings	1	1	0	1
Mobile phones, airtime, and data bundles	24	14	31	24
Biomarker equipment, reagents, and supplies	6	2	3	11
Overhead	9	9	9	9

Abbreviations: MNH, maternal and newborn health; n/a, not available.

## DISCUSSION

The MNH eCohort is a long and complex tool that requires time and infrastructure to administer. The progress to date in Ethiopia, India, Kenya, and South Africa serves as proof of concept that the MNH eCohort is a feasible approach to gather rich, robust, and actionable data to improve MNH services. The eCohort is not an intervention itself but a survey tool meant to assess health system quality. This methodology is particularly well suited to gather policy-relevant information on health system continuity, particular health conditions that arise in the prenatal and postnatal periods, and women’s experience of care—and how it changes—over the continuum of maternal health care.

The progress to date in Ethiopia, India, Kenya, and South Africa serves as proof of concept that the MNH eCohort is a feasible approach to gather rich, robust, and actionable data to improve MNH services.

While the MNH eCohorts are in the final stages of data collection, some analysis has already begun with available data, and the findings are useful to program managers and policymakers alike. The development of policy briefs that describe key measures of service and system competence across the continuum of MNH care is in progress and should inform improvements for MNH service quality and health outcomes.^15^ Ethiopia and Kenya have begun the analysis of their data and have drafted policy briefs that highlight important findings for program managers and policymakers. In Ethiopia, the eCohort showed that during the first ANC visit, 95% of women had blood tests, 86% of women had a urine test, but only 45% of women had an ultrasound, and only 11% of women were screened for malnutrition. The limited screening of malnutrition is an important area of improvement for Ethiopia’s Ministry of Health, and these findings, particularly as more data come in, can help identify bottlenecks and action points. In Kenya, women with risk factors received the same amount of care as women without any risk, and women who reported danger signs received the same amount of care as women not reporting danger signs. These findings highlight a need for better-targeted management of women at risk of poor pregnancy outcomes. These learnings are important and actionable to improve quality of care during the MNH continuum.

There are some limitations to the eCohort methodology, notably that it is a long survey with many data collection touchpoints. Data collection teams reported that the monthly phone call frequency may have been excessive and required full-time teams to perform calls for the 1,000 women enrolled. In Ethiopia, data collectors reported being able to conduct only 6 follow-up surveys per day. The maximum time that women were willing to spend on the phone was around 25–30 minutes. Another limitation is that routine follow-up of women may modify behavior and may entice them to seek more care than they would have if they had not been enrolled in the eCohort. Providers may also modify their behavior if they are aware that women are enrolled in the study. It is important to note that quality of care in these settings is often poor. Therefore, this study is unlikely to substantially improve the quality of care that women receive. Additionally, the study may only impact provider behavior for the first ANC visit because women may attend ANC or deliver at other health facilities where providers do not know that they are enrolled in the eCohort study.

To be successfully implemented, the MNH eCohort requires (1) a carefully reviewed tool that is reflective of national programs and contexts; (2) accurate programming of all modules on a data collection software; (3) capable and reliable data collectors with data collection experience and understanding of MNH; and (4) experience with phone-based data collection, including infrastructure to carry out this component of the study.

Few studies currently assess longitudinal MNH care quality in LMICs. While the comparison of the MNH eCohort to other tools is described in detail elsewhere, this methodology is unique, given the longitudinal nature of data collection. Other surveys, including the Performance Monitoring for Action Longitudinal Survey Panel, share some similar characteristics with the eCohorts, but the eCohorts provide unique information on undermeasured dimensions of health system quality. The eCohort talks to women monthly throughout their pregnancy and postpartum period. The eCohort measures important components of care competence—for care delivered at the right time, according to national and global guidelines. The eCohort also measures system competence: what happens between visits, whether care is continuous and integrated, and the ability to detect and treat conditions appropriately. These measures of care and system competence are not routinely available in existing data and provide important policy-relevant findings to improve system quality and health outcomes. Other surveys that assess MNH care quality are mostly cross-sectional, do not follow women after they leave the facility, and tend to focus on providers and the facility, not the clients themselves.^16^

The eCohort is a long, complex survey (follow-up lasts almost a year if women initiate ANC early in their first trimester). While the data are rich, we realize this may feel too cumbersome for many potential users of the data. Our team will be analyzing survey data to determine ways to shorten the follow-up time, reduce some measures, and assess other ways of making the eCohort a less intensive instrument.

Already, the eCohort methodology is being adapted and used for conditions beyond MNH, including for chronic conditions such as hypertension in Latin America. This methodology has also been adapted as an evaluative tool to assess program performance in Mexico. Future adaptations of the MNH eCohort will include a standardized and preprogrammed set of tools on SurveyCTO for easier implementation. Other adaptations might include an eCohort that is (1) scalable and national representative, (2) can be implemented in a shorter period, and/or (3) lighter by focusing on a smaller set of core indicators.

The application of longitudinal data collection, with a user focus, in diverse sentinel sites will contribute rich learnings on health system performance across the MNH continuum of care. The eCohorts will produce learnings related to novel dimensions of quality, including care competence, system competence, and user experience, and, to our knowledge, is the only tool gathering such information.

## Notes

**Peer Reviewed**

**First Published Online:** August 20, 2024.
